# Radical cyberknife radiosurgery with tumor tracking: an effective treatment for inoperable small peripheral stage I non-small cell lung cancer

**DOI:** 10.1186/1756-8722-2-1

**Published:** 2009-01-17

**Authors:** Brian T Collins, Saloomeh Vahdat, Kelly Erickson, Sean P Collins, Simeng Suy, Xia Yu, Ying Zhang, Deepa Subramaniam, Cristina A Reichner, Ismet Sarikaya, Giuseppe Esposito, Shadi Yousefi, Carlos Jamis-Dow, Filip Banovac, Eric D Anderson

**Affiliations:** 1 Department of Radiation Medicine, Georgetown University Hospital, Washington, DC, USA; 2 Biostatistics Unit, Lombardi Comprehensive Cancer Center, Georgetown University, Medical Center, Washington, DC, USA; 3 Department of Hematology and Oncology, Georgetown University Hospital, Washington, DC, USA; 4 Division of Pulmonary, Critical Care and Sleep Medicine, Georgetown University Hospital, Washington, DC, USA; 5 Department of Nuclear Medicine, Georgetown University Hospital, Washington, DC, USA; 6 Department of Radiology, Georgetown University Hospital, Washington, DC, USA; 7 Division of Vascular & Interventional Radiology, Georgetown University Hospital, Washington, DC, USA

## Abstract

**Objective:**

Curative surgery is not an option for many patients with clinical stage I non-small-cell lung carcinoma (NSCLC), but radical radiosurgery may be effective.

**Methods:**

Inoperable patients with small peripheral clinical stage I NSCLC were enrolled in this study. Three-to-five fiducial markers were implanted in or near tumors under CT guidance. Gross tumor volumes (GTVs) were contoured using lung windows. The GTV margin was expanded by 5 mm to establish the planning treatment volume (PTV). A dose of 42–60 Gy was delivered to the PTV in 3 equal fractions in less than 2 weeks using the CyberKnife radiosurgery system. The 30-Gy isodose contour extended at least 1 cm from the GTV. Physical examination, CT imaging and pulmonary function testing were completed at 6 months intervals for three years following treatment.

**Results:**

Twenty patients with an average maximum tumor diameter of 2.2 cm (range, 1.1 – 3.5 cm) and a mean FEV1 of 1.08 liters (range, 0.53 – 1.71 L) were treated. Pneumothorax requiring tube thoracostomy occurred following CT-guided fiducial placement in 25% of the patients. All patients completed treatment with few acute side effects and no procedure-related mortality. Transient chest wall discomfort developed in 8 of the 12 patients with lesions within 5 mm of the pleura. The mean percentage of the total lung volume receiving a minimum of 15 Gy was 7.3% (range, 2.4% to 11.3%). One patient who received concurrent gefitinib developed short-lived, grade III radiation pneumonitis. The mean percent predicted DLCO decreased by 9% and 11% at 6 and 12 months, respectively. There were no local failures, regional lymph node recurrences or distant metastases. With a median follow-up of 25 months for the surviving patients, Kaplan-Meier overall survival estimate at 2 years was 87%, with deaths due to COPD progression.

**Conclusion:**

Radical CyberKnife radiosurgery is a well-tolerated treatment option for inoperable patients with small, peripheral stage I NSCLC. Effective doses and adequate margins are likely to have contributed to the optimal early local control seen in this study.

## Background

Standard therapy for operable clinical stage I non-small cell lung cancer (NSCLC) is lobectomy, a radical surgery requiring complete removal of the involved lobe plus ipsilateral hilar and mediastinal lymph node dissection.[1] Tumor recurrence is infrequent following lobectomy and limited to the regional lymph nodes or distant sites. However, despite recent improvements,[2] lobectomy remains a major operation associated with early mortality,[3] a decline in pulmonary function[1] and multiple postoperative morbidities.[4] Recently, sublobar resection with adequate margins (> 1 cm) has been advocated for marginally operable patients with small peripheral lesions.[5] Such treatment in appropriately selected patients provides excellent local control without the early mortality and significant decline in lung function associated with lobectomy.

Treatment options for patients with clinical stage I NSCLC who are not surgical candidates are limited. Inferior outcomes with conventionally fractionated radiation approaches have been largely attributed to poor local tumor control.[6] secondary to historically necessary prolonged treatment courses, which diminish the effectiveness of the therapy.[7,8] The development of the stereotactic body frame with abdominal compression to dampen respiratory lung motion has allowed for the treatment of small mobile peripheral lesions with comparatively tight margins (1 cm) on the gross tumor.[9] Recently completed trials suggest that extremely high biologically effective doses may be delivered safely and rapidly to small peripheral lung tumors with this enhanced accuracy. [10-12] As anticipated, such treatment has resulted in improved early local control rates.[13]

We began treating small peripheral lung tumors in mid-2004 using the CyberKnife^® ^frameless robotic radiosurgery system (Accuray Incorporated, Sunnyvale, CA) with Synchrony^® ^respiratory motion tracking.[14,15] The accuracy and flexibility of the system allowed us to deliver dose distributions capable of eradicating both the gross tumor and the microscopic disease radiating from it.[16,17] The goal was similar to that of sublobar resection, i.e., to eliminate the tumor with 1 cm or greater margins, and thus the approach was designated radical radiosurgery.[14] We report preliminary outcomes from 20 consecutive inoperable patients with small, peripheral, clinical stage I NSCLC treated using this novel treatment approach.

## Materials and methods

### Eligibility

The Georgetown University Hospital institutional review board approved this study and all participants provided informed written consent. The multidisciplinary thoracic oncology team evaluated patients. Prior to treatment, CT imaging of the chest, abdomen and pelvis with IV contrast, PET imaging, and routine pulmonary function tests (PFTs) were completed. Inoperable patients with pathologically confirmed small, peripheral, clinical Stage I NSCLC were treated. Inoperability was defined as a post-operative predicted forced expiratory volume in one second (FEV1) of less than 40%, post-operative predicted carbon monoxide diffusing capacity (DLCO) of less than 40%, VO_2 _max less than 10 ml/kg/min, or severe comorbid medical conditions.[18] Tumors were considered small if the maximum diameter and gross volume measured less than 4 cm and 30 cc, respectively. Tumors were considered peripheral if radical treatment was feasible without exceeding predetermined critical central structure maximum point dose limits (Table 1).

**Table 1:** Central Critical Structure Radiation Dose Limits

**Adjacent Structure**	**Maximum Dose Limit (total for 3 fractions)**
Spinal cord	18 Gy

Left ventricle	18 Gy

Esophagus	24 Gy

Main bronchus	30 Gy

Trachea	30 Gy

Aorta	40 Gy

### Fiducial Placement

With conscious sedation and local anesthesia, 3 to 5 gold fiducials measuring 0.8–1 mm in diameter by 3–7 mm in length (Item 351-1 Best Medical International, Inc., Springfield, VA) were placed with adequate spacing (1–2 cm) in or near tumors under CT-guidance as previously described.[19,20]

### Treatment Planning

Fine-cut (1.25-mm) treatment planning CTs were obtained 7–10 days after fiducial placement during a full inhalation breath-hold. Gross tumor volumes (GTV) were contoured utilizing lung windows. The GTV margin was expanded 5 mm to establish the planning treatment volume (PTV). All critical central thoracic structures (Table 1) and the lungs were contoured to ensure that incidental radiation delivered to these structures was limited. A treatment plan was generated using the CyberKnife non-isocentric, inverse-planning algorithm with tissue density heterogeneity corrections for lung. No attempt was made to treat at-risk but clinically negative lymph nodes (elective nodal irradiation). In general, lower doses within the radical range of 42 to 60 Gy in 3 fractions were prescribed when concerns about adjacent critical structures arose and when patients were felt to have severe pulmonary dysfunction. The radiation was delivered to an isodose line that covered at least 95% of the PTV and resulted in the 30-Gy isodose contour extending a minimum of 1 cm from the GTV. The percentage of the total lung volume receiving 15 Gy or more (V15) was limited to 15%. Finally, treatments were designed to be deliverable in 2 hours or less.

### Treatment Delivery

Patients were treated according to the Georgetown University Hospital small peripheral pulmonary nodule protocol as previously described.[14] Briefly, pretreatment fluoroscopy confirmed that fiducial motion correlated with tumor motion. Subsequently, patients were brought to the CyberKnife suite and laid supine on the treatment table with their arms at their side. Three red light-emitting diodes (LEDs) were placed on the patient's anterior torso directed toward the camera array. Fiducials were located using the orthogonal x-ray imagers. A correlation model was created between the LEDs tracked continuously by the camera array and the fiducial positions imaged periodically by the x-ray targeting system. During treatment delivery the tumor position was tracked using the live camera array signal and correlation model; the linear accelerator was moved by the robotic arm to maintain precise alignment with the tumor throughout the respiratory cycle. Fiducials were imaged prior to the delivery of every third beam to verify targeting accuracy and to update the correlation model.

### Follow-up Studies

Physical examination, CT imaging and routine PFTs were performed at 6-month intervals. Complete response was defined as resolution of the tumor on CT imaging and partial response as a decrease in the tumor volume relative to the treatment planning CT. Local tumor and regional lymph node recurrence was defined as unequivocal progression on serial CT imaging. Biopsy was required to confirm recurrence. Toxicity was scored according to the National Cancer Institute Common Terminology Criteria for Adverse Events, Version 3.0.[21]

### Statistical Analysis

The follow-up duration was defined as the time from the date of completion of CyberKnife treatment to the last date of follow-up or the date of death. Actuarial survival and local control were calculated using the Kaplan-Meier method. Two-sided Wilcoxon signed-rank tests were used to assess the statistical significance of changes in pulmonary function tests following radiosurgery; which were determined using an alpha level of 0.05. Post CyberKnife treatment changes in percent predicted FEV1, DLCO and total lung capacity (TLC) were evaluated at 6, 12, 18 and 24 months.

## Results

### Patient and Tumor Characteristics

Twenty consecutive patients (5 men and 15 women) with inoperable clinical stage I NSCLC (adenocarcinoma 10, NSCLC not otherwise specified 7 and squamous cell carcinoma 3) and an Eastern Cooperative Oncology Group (ECOG) performance status of 2 or less were treated over a 30-month period extending from October 2004 to April 2007 (Table 2). The median follow-up time among survivors was 25 months (range, 6–36 months). No patients were lost to follow-up. All patients were heavy smokers, 80% of whom had stopped smoking in the distant past (> 3 years). Two patients chose to continue smoking despite being diagnosed with lung cancer. Pulmonary dysfunction was the primary rationale for non-surgical treatment and 5 patients required supplemental oxygen prior to enrollment. Three patients were denied surgical treatment based solely on cardiac insufficiency. Sixty percent of the lesions involved the upper and middle lobes. The mean maximum tumor diameter was 2.2 cm (range, 1.1 – 3.5 cm) and the mean GTV was 9.7 cc (range, 1.3 – 24.4 cc).

**Table 2:** Patient and Tumor Characteristics

	**Mean (Range)**
Age (years)	74 (64 – 86)

Weight (lbs)	156 (116 – 225)

FEV1 (L)	1.08 (0.53 – 1.71)

% predicted FEV1	52 (21 – 84)

% predicted TLC	103 (69 – 136)

% predicted DLCO	57 (44 – 83)

Maximum Tumor Diameter (cm)	2.2 (1.1 – 3.5)

Gross Tumor Volume (cc)	9.7 (1.3 – 24.4)

### Treatment Characteristics

Treatment plans were composed of hundreds of pencil beams shaped using a single 20, 25, 30 or 35-mm diameter circular collimator (Table 3). An average of 53 Gy was delivered to the prescription isodose line in three 1–2 hour treatments over a 5 to 11 day period (mean, 7 days). The percentage of the total lung volume receiving 15 Gy or more was low (range, 2.8 – 11.3%) despite the radical treatment intent. On average, 53 paired orthogonal x-ray images of the fiducials were taken during each treatment to confirm the accuracy of the correlation model. Two patients received concurrent systemic therapy as prescribed by their treating oncologists. One of these patients completed treatment flanked by cycles 2 and 3 of full-dose carboplatin and docetaxel. The second patient received concurrent gefitinib.

**Table 3:** Treatment Characteristics

	**Mean (Range)**
Prescribed Dose (Gy)	53 (42 – 60)

Biologic Effective Tumor Dose (BED Gy_10_)	147 (100–180)

Prescription Isodose Line (%)	81 (75 – 85)

Planning treatment volume coverage (%)	99 (95 – 100)

Number of beams per fraction	156 (79 – 242)

Number of paired x-ray verification images per fraction	52 (26 – 81)

Beam-on time (minutes)	73 (54–124)

Treatment course (days)	7 (5–11)

% Total lung volume receiving 15 Gy or more	7.3 (2.8–11.3)

### Complications

Pneumothorax requiring tube thoracostomy developed in 25% of patients following fiducial placement. Subsequently, all patients completed treatment without interruption or noteworthy side effects. Following treatment, acute toxicity consisting of mild transient fatigue was reported in the majority of patients. Chest wall discomfort, typically lasting several weeks, developed in 8 of 12 patients with tumors in close proximity to the pleura (5 mm). Classic acute grade III radiation pneumonitis was observed in 1 patient who had received 60 Gy with concurrent gefitinib treatment. Despite her relatively good lung function (FEV1 = 1.51 L), small GTV (7.56 cc) and low V15 (9.5% of total lung volume), she developed an infiltrate corresponding with the high dose treatment volume and hypoxia requiring supplemental oxygen 4 weeks following CyberKnife treatment. Her acute symptoms appeared unrelated to her severe underlying heart disease and resolved with steroids. She discontinued gefitinib and is well two years following treatment.

### Post-treatment Pulmonary Status

Among the entire group, no statistically significant change was seen in percent predicted FEV1 and TLC at 6, 12, 18 or 24 months. Statistically significant reductions of 9% (from 57% to 48%; p = 0.005) and 11% (from 57% to 46%; p = 0.05) in the mean percent predicted DLCO were seen at 6 and 12 months, respectively. Reductions in DLCO at 18 and 24 months did not reach statistical significance.

### CT Tumor Response

**Figure 1:**
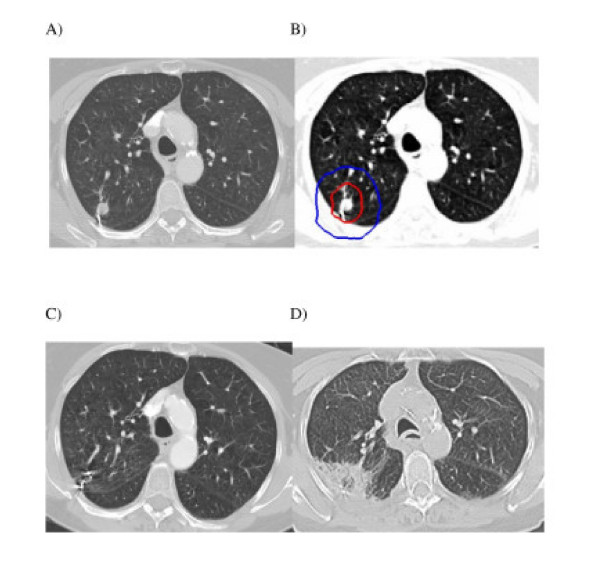
**Right upper lobe clinical stage IA NSCLC treatment planning CT (A), planned radiation dose distribution (B: the planning treatment volume is shown in red and the 30 Gy isodose line in blue), and CT at 3 and 6 months post-treatment (C and D) show an initial decrease in the tumor volume followed by radiation fibrosis which correlates with the planned dose distribution, engulfs the fiducials and impedes evaluation of tumor response**.

Six-month CT scans were available for all 20 patients. Thirteen lesions responded to treatment as documented by a decrease in tumor volume. Seven lesions were obscured by radiation fibrosis at 6 months. At 12 months, 16 patients' CT scans were available for review. Eight lesions continued to respond to treatment, three of which had resolved completely. Eight lesions were obscured by radiation fibrosis at 12 months. At 18 months, 12 patients' CT scans were available for review. Two lesions responded completely, 2 exhibited a partial response to treatment with only minimal residual soft tissue abnormality remaining, and 8 were completely obscured by radiation fibrosis. In each case fibrosis corresponded with the planned high-dose treatment volume and uniformly encompassed the fiducials (Figure 1). There were no major changes in tumor response following the 18 month evaluation (Table 4). Serial imaging characteristics suggesting local failure were not observed during early follow-up and consequently no confirmatory biopsies have been completed.

**Table 4:** Tumor response per CT imaging

	**6 months (%)**	**12 months (%)**	**18 Months (%)**
Complete Response	0	19	17

Partial Response	65	31	17

Obscured by Fibrosis	35	50	66

### Disease Spread and Survival

**Figure 2:**
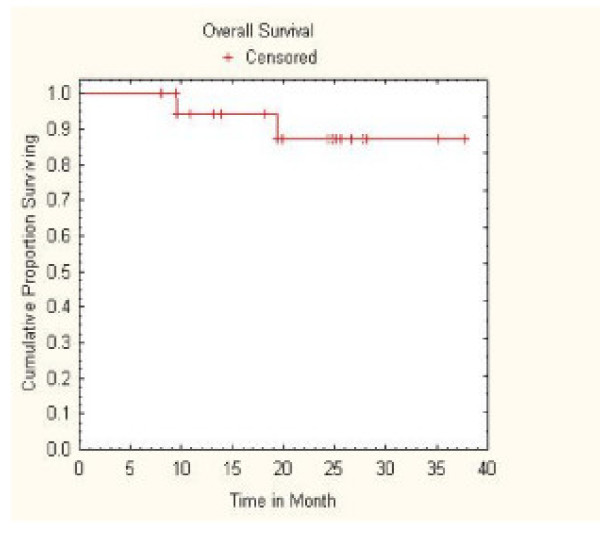
**Kaplan-Meier plot of overall survival**.

No regional lymph node failures or distant metastases were observed during early follow-up. However, two oxygen-dependent patients with pre-treatment FEV1 values of 0.53 and 0.76 liters died of progressive lung dysfunction at 9 and 18 months, respectively. Therefore, with a median follow-up of 25 months for surviving patients, Kaplan-Meier overall survival at 2 years was 87% (Figure 2).

## Discussion

Stage I NSCLC is curable.[22] Peripheral lung tumors are more likely to be cured with radiosurgery than central cancers because there is less untreated lymphatic spread[23] and a more favorable therapeutic window.[11] However, consistently curing these patients without surgery will require adequate gross tumor doses with finely tailored dose gradients capable of eradicating known relatively radiation-sensitive microscopic tumor extensions, while adequately preserving lung function.

In late 2004, we initiated a radical CyberKnife protocol for medically inoperable patients with small, peripheral, stage I NSCLC. Ultimately, we treated a select group of patients with relatively good performance status and small tumor volumes because we were concerned about CT-guided fiducial placement and treatment-related pulmonary toxicity. Mandatory minimum gross (42 Gy)[24] and microscopic tumor doses (30 Gy) [25-27] were derived from historical clinical data. Continuous tracking of respiratory tumor motion and highly accurate beam alignment throughout treatment with the CyberKnife allowed us to deliver radical dose distributions with tighter margins on the GTV than historically feasible (5 mm).[14] Numerous pencil beams were used to produce dose gradients that conform closely to the shape of the target, resulting in theoretically adequate microscopic disease doses extending an ample 1 cm or more from the tumor.[28] Twenty patients have been treated in 30 months. With a median follow-up of 25 months for surviving patients, the 2-year Kaplan-Meier local control rate was 100%, the 2-year Kaplan-Meier overall survival rate was 87%, and there have been no severe (grade IV) treatment-related complications or early mortalities. Furthermore, despite the comprehensive nature of the treatment, the decrement in lung function remained at acceptable levels. Thus, we conclude that radical radiosurgery with real-time tumor motion tracking using the CyberKnife is a safe and effective treatment option for small peripheral Stage I NSCLC.

Despite promising preliminary results, critical issues concerning the evaluation of treatment efficacy and selection of patients merit additional consideration. Radiosurgery delivered to small peripheral tumors with margins adequate to treat radial microscopic extension (> 1 cm) will damage peritumoral lung tissue. The acute lung injury will often result in asymptomatic focal lung parenchyma fibrosis corresponding with the high-dose radiation volume. [29-32] In the present study all tumors initially responded to treatment with a decrease in volume on CT. However, by the six-month mandatory evaluation, all patients had developed CT evidence of peritumoral radiation fibrosis. At 18 months, two thirds of the tumors were obscured by such fibrosis, making CT tumor assessment difficult. Preliminary analysis of PET imaging suggests that it too is unreliable following radiosurgery.[33] Although this trial did not require PET/CT imaging, it was routinely completed. Moderate PET activity was often observed following treatment, but could uniformly be attributed to radiation fibrosis rather than tumor progression on serial imaging. Until a dependable noninvasive means to identify early local recurrence in the presence of fibrosis is developed, inoperable patients with fibrosis will require close follow-up which may include biopsy.[31]

In late 2003, Timmerman et al. published preliminary results of the Indiana University inoperable stage I NSCLC SBRT dose escalation trial.[12] The primary finding of this study was that 60 Gy in 3 fractions could be safely delivered to inoperable stage I NSCLC patients in less than 2 weeks if relatively tight gross tumor margins (1 cm) were used. However, prior to proceeding with phase II studies, maturing data suggested that critical central thoracic structures tolerated high-dose hypofractionated radiation poorly. Accordingly, the Radiation Therapy Oncology Group (RTOG) protocol 0236 was limited to small (< 5 cm) tumors that lay outside the central bronchial tree, a large area that extends 2 cm from the major airways.[11] We chose to deliver a range of radical doses (42–60 Gy) to smaller tumors (< 4 cm) with tighter margins (5 mm) than RTOG 0236. Therefore, we felt confident selecting a more inclusive definition of peripheral tumor as those tumors located a sufficient distance from sensitive critical central thoracic structures so that radical radiation doses could be delivered to the PTV while adhering to maximum point dose limits (Table 1). To date, with sufficient follow-up to detect late radiation toxicity, clinically apparent radiation damage to critical central structures has not been observed despite our delivery of a mean dose of 53 Gy to the PTV.

Peripheral thoracic structures such as the lung parenchyma and the chest wall did sustain clinically apparent damage as anticipated. Despite the radical treatment intent, the mean percentage of the total lung volume receiving a minimum of 15 Gy was only 7.3% (range, 2.4% to 11.3%). Nonetheless, acute Grade III radiation pneumonitis occurred in a single patient treated with concurrent gefitinib, an alleged potentiator of radiation-induced lung fibrosis.[34] Although it is tempting to further limit the percentage of lung receiving greater than 15 Gy in an attempt to decrease the risk of radiation pneumonitis, this should only be considered if the radical treatment intent is maintained.

All evaluated patients were heavy smokers, 80% of whom had stopped smoking in the distant past (> 3 years). Therefore, although the baseline pulmonary function was poor, PFTs just prior to and following the treatment were largely unaffected by recent smoking. The treatment did not adversely affect FEV1 or TLC; however, it did cause significant early reductions in the mean percent predicted DLCO. Predictably, the magnitude of this decline was small, as it was in a recently reported segmentectomy series.[35] Regrettably, 2 patients with severe COPD, who required continuous supplemental oxygen prior to treatment, died 9 and 18 months after radiosurgery secondary to progressive pulmonary dysfunction. It is unknown whether their deaths were premature and attributable to treatment. Nonetheless, it is possible that a population of inoperable stage I NSCLC patients exists, possibly those whom are oxygen dependent, who may not benefit from radical treatment. Such patients may benefit from a more conservative radiosurgery approach with lower doses [36,37] or tighter margins.[38]

Thoracic surgery uniformly results in permanent chest wall scarring and acute pain, which may persist. Nonetheless, it remains the standard treatment for stage I NSCLC. In contrast, carefully designed early stage lung cancer radiosurgery may result in only trivial radiation skin reactions and transient, mild to moderate chest wall pain. The use of hundreds of lightly weighted beams in this study rather than a few heavily weighted ones has prevented the infrequent but potentially severe skin injuries reported in prior thoracic radiosurgery series conducted using other radiosurgical instruments.[12,39] However, mild-to-moderate chest wall pain, typically lasting several weeks, was seen following treatment in the majority of patients with lesions within 5 mm of the pleura. Limiting the dose to the chest wall would likely diminish the severity of this complication; however, this may have led to potentially life threatening local failures and therefore is not recommended at this time given the acceptable and transient nature of the toxicity observed to date.

The current study required CT-guided fiducial implantation prior to treatment. This procedure frequently results in pneumothorax in high-risk patients, often necessitating tube thoracostomy and a short hospital stay.[37] Fortunately, alternative fiducial placement approaches and a fiducial-free peripheral lung tumor tracking system are now available. [40-42] Ongoing research will determine appropriate patient selection for these new approaches and their efficacy. In the meantime, fiducial tracking and CT-guided fiducial placement will remain our standard approach for small, peripheral lung tumors. The risk of pneumothorax is justified by the optimal intrafraction targeting verification made possible by properly placed fiducials. We have found that frequent intrafraction targeting verification and continuous tumor tracking with the Synchrony system allows the delivery of adequate dose to the gross tumor and its microscopic extension while keeping the volume of healthy lung exposed to radiation at safe levels. Although there are other ways of dealing with the problem of treating tumors that move with respiration, our experience has made us confident of the safety and accuracy of this approach.

## Conclusion

Thoracic radiosurgery is a new treatment option for stage I NSCLC.[13,24,39] Optimal clinical outcomes will require proper patient selection and adequate radiation doses. Inoperable oxygen-independent patients with small peripheral lung tumors are ideal candidates.[11] The delivery of hundreds of radiation beams while continuously tracking and compensating for respiratory tumor motion will ensure that the gross tumor and radial microscopic extension are effectively treated. Our early experience suggests that radical CyberKnife radiosurgery will result in durable local control with acceptable toxicity. However, larger studies with pathologic confirmation of tumor eradication and ample follow-up are needed to confirm our preliminary findings. At this time, surgery remains the standard treatment option for operable patients with stage I NSCLC.

## Abbreviations

BED Gy_10_: biologic effective tumor dose; CT: computed tomography; DLCO: diffusing capacity of the lung for carbon monoxide; FEV1: forced expiratory volume in 1 sec; GTV: gross tumor volume; Gy: Gray; NSCLC: non-small cell lung cancer; PET: positron emission tomography; PFT: pulmonary function tests; PTV: planning treatment volume; TLC: total lung capacity; V15: total lung volume receiving 15 Gy or more.

## Competing interests

BC is an Accuray clinical consultant. EA is paid by Accuray to give lectures.

## Authors' contributions

BC drafted the manuscript, participated in treatment planning, data collection and data analysis. SV participated in data collection, data analysis and manuscript revision. KE participated in data collection, data analysis and manuscript revision. SC prepared the manuscript for submission, participated in data collection, data analysis and manuscript revision. SS created tables and figures and participated in data analysis and manuscript revision. XY participated in treatment planning, data collection and data analysis. YZ performed the biostatistical analysis and created figures. DS participated in data analysis and manuscript revision. CR participated in data analysis and manuscript revision. IS participated in data collection, data analysis and manuscript revision. GE participated in data collection, data analysis and manuscript revision. SY participated in data analysis and manuscript revision. CJ participated in treatment planning, data analysis and manuscript revision. PB participated in treatment planning, data collection, data analysis and manuscript revision. EA participated in treatment planning, data collection, data analysis and manuscript revision
